# A real-world pharmacovigilance study of FDA Adverse Event Reporting System (FAERS) events for Definity

**DOI:** 10.1371/journal.pone.0331444

**Published:** 2025-08-29

**Authors:** Wanting Jiang, Shudan Yin, Lingmin Li, Menghao Teng

**Affiliations:** 1 Department of Ultrasound Imaging, Xi’an People’s Hospital (Xi’an Fourth Hospital), Xi’an, Shaanxi, China; 2 Department of Sports Medicine and Joint Surgery, Xi’an Ninth Hospital), Xi’an, Shaanxi, China; Auburn University, UNITED STATES OF AMERICA

## Abstract

**Background:**

Definity significantly enhances the diagnostic accuracy of echocardiography but raises ongoing safety concerns. This study aimed to explore potential adverse events (AEs) associated with Definity in real-world settings by analyzing data from the U.S. Food and Drug Administration (FDA) Adverse Event Reporting System (FAERS).

**Methods:**

We retrospectively extracted AE reports from the FAERS database between 2004 and the first quarter of 2024. Disproportionality analyses using reporting odds ratio (ROR), proportional reporting ratio (PRR), Bayesian Confidence Propagation Neural Network (BCPNN), and multi-item gamma Poisson shrinker (MGPS) were conducted to identify signals potentially associated with Definity. Univariate and multivariate logistic regression analyses were performed as sensitivity analyses to assess potential risk factors for Definity-related AEs.

**Results:**

A total of 4460 reports with Definity as the “primary suspected” drug were included. We identified 104 statistically significant signals at PT level, including common AEs such as back pain and muscle spasms, as well as exploratory signals not listed on the drug label, such as eye movement disorder and renal pain. It is noteworthy that 21 signals did not retain significance following Bonferroni correction. These AEs spanned 25 System Organ Classes (SOCs). In reports with available time-to-onset data, most events occurred within 30 days of administration, while some were reported at longer intervals. Moreover, logistic regression analysis indicated that both gender and body weight were independent risk factors associated with Definity-related AE.

**Conclusion:**

This study provides exploratory insights into potential AEs associated with Definity based on real-world pharmacovigilance data. While most signals are consistent with known safety profiles, several emerging signals warrant further investigation and clinical awareness. These findings may contribute to ongoing risk management and pharmacovigilance efforts.

## 1. Introduction

Echocardiography is a safe, portable, and noninvasive technique used to evaluate heart structure and function, both at rest and during stress [1]. However, around 15% of patients pose technical challenges for this imaging technique due to factors like obesity, lung disease, or poor physical condition [2]. To overcome these limitations, echocardiographic contrast agents (ECAs) were developed [3]. Definity, a second-generation ECA available in the United States, is a perflutren lipid microsphere composed of microbubbles ranging from 1.1 to 3.3 um in diameter [4]. Each microbubble has a phospholipid shell encapsulating fluorocarbon gas. Definity’s components are biologically inert, mimicking the rheological properties of red blood cells in the bloodstream, and its excellent ultrasound scattering properties enhance the distinction between blood pools and the endocardium [4]. Approved by the U.S. Food and Drug Administration (FDA) in June 2001, Definity is recommended for patients with suboptimal echocardiograms to opacify the left ventricular chamber and improve visualization of the left ventricular endocardial border [5,6]. Early studies have been demonstrated that Definity significantly improves the accuracy of assessing left ventricular volumes and ejection fraction, aids in detecting left ventricular thrombus, and enhances the diagnostic precision of stress echocardiography [7]. Although there are data showing that Definity is underutilized in clinical practice, its usage rate is increasing year by year [8].

The safety of Definity has been evaluated in clinical trials, however, due to the strict inclusion criteria and relatively limited sample size of the trial population, it was not possible to fully identify rare serious adverse events (AE) during the clinical trial phase. With the widespread use of echocardiography in hospitals, the safety of Definity has drawn increasing attention. In October 2007, after receiving spontaneous reports of 4 deaths and 190 serious cardiopulmonary reactions related to ECAs from healthcare providers, the FDA updated Definity’s safety information, prohibiting its use in patients with pulmonary hypertension or unstable cardiopulmonary conditions [9]. Earlier post-marketing studies on the safety of Definity have indicated that life-threatening adverse drug events (ADEs) are extremely rare, with an incidence of less than 1 in 10,000 [10,11]. These reactions share some clinical features with anaphylaxis but are not IgE-mediated, instead, they may occur even in individuals who have never been exposed to Definity before, therefore, they are classified as Type I hypersensitivity reactions, also known as complement activation-related pseudoallergy [12,13]. In 2021, the FDA announced 11 cases of allergic reactions in patients with polyethylene glycol allergies triggered by ultrasound contrast agents, including Definity [14,15]. Since no data indicated a widespread increase in the frequency of ADEs, no major revisions were made to Definity’s indications at that time [14,15]. However, in recent years, several case reports have suggested the potential of Definity to induce vaso-occlusive crises [16,17]. Therefore, it remains necessary to further evaluate the safety of Definity to detect emerging serious ADEs.

The FDA Adverse Event Reporting System (FAERS) is one of the largest databases for gathering and evaluating ADEs associated with drug usage worldwide [18,19]. These publicly accessible data provide clinicians with a crucial resource for evaluating medication safety. Updated quarterly, the database covers AE reports from a variety of sources, such as healthcare professionals, patients, and pharmaceutical companies. These reports encompass AEs related to drug use, medication errors, drug abuse, and other drug-related incidents, and are summarized on the FDA’s website [20]. Given the lack of evidence of AEs for Definity at the real-world level, we utilized the FAERS database to analyze AEs linked to Definity from the first quarter of 2004 to the first quarter of 2024. This study aims to identify novel risk signals associated with Definity and determine the time interval for their onset, thereby enhancing pharmacovigilance efforts and improving clinical awareness. Our findings may help raise awareness of previously under-recognized ADEs, such as pain-related events and hypersensitivity reactions, and serve as a useful reference for prescribers, patients, and regulatory authorities. By highlighting these signals, this study may contribute to future updates in product labeling and clinical practice recommendations.

## 2. Methods

### 2.1 Data source and preprocessing

**Fig 1:**
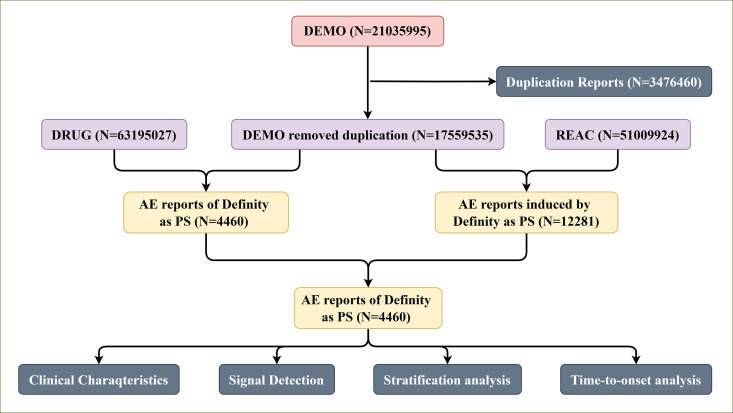
The flowchart of the whole study.

In this study, we extracted the datasets from the FAERS database, covering the period from the first quarter of 2004 to the first quarter of 2024. The collected FAERS datasets have seven files: demographic and administrative information (DEMO), adverse drug reaction information (REAC), patient outcome information (OUTC), drug information (DRUG), drug therapy starts dates and end dates (THER), information on report sources (RPSR), and indications for use/diagnosis (INDI). Given the dynamic nature of data updates, duplicate reports are inevitable in the FAERS database. To improve the reliability of our results, we removed duplicates based on FDA-recommended criteria [21,22]. Specifically, when the CASEID was the same, we retained the record with the most recent FDA_DT. If both CASEID and FDA_DT were identical, we retained the record with the higher PRIMARYID [23]. After preprocessing, 17,559,535 DEMO reports, 63,195,027 DRUG cases, and 51,009,924 REAC records were obtained (Fig 1).

### 2.2 Drug selection

Considering that there was no a uniform name about Definity in FAERS database, we gathered all names associated with Definity from the NCBI MeSH database. This included generic and brand names, chemical names, and various synonyms of its active ingredient, to ensure comprehensive coverage of all its naming conventions. The complete list of searched drug names is provided in S1 Table and was used to identify ADEs related to Definity. Moreover, drug usage in the FAERS database is classified into four categories: primary suspect (PS), second suspect (SS), concomitant (C), and interacting (I) [24]. We included only case reports in which Definity was identified as the PS. As a result, a total of 4460 ADE reports related to Definity administration were identified.

### 2.3 Data standardization

The Medical Dictionary for Regulatory Activities (MedDRA) was used in this study to standardize AE data to ensure consistency across records [25]. MedDRA is a standardized medical terminology system with a hierarchical structure comprising five levels, from lowest to highest: lowest-level term (LLT), preferred term (PT), high-level term (HLT), high-level group term (HLGT), and system organ class (SOC). All ADEs were coded using PTs and subsequently mapped to their corresponding highest SOCs using MedDRA version 26.0 [24]. To minimize confounding due to indication bias, PTs that clearly reflected the intended diagnostic use or known clinical indications for Definity (e.g., “echocardiogram”, “left ventricular dysfunction”) were excluded from the signal detection analysis, based on product label information, literature review, and expert judgment [7]. While SOC terms were used descriptively to categorize and summarize the distribution of AEs, all disproportionality analyses were strictly conducted at the PT level to ensure methodological rigor and clinical specificity. In conclusion, 12281 PTs associated with Definity as the PS (Definity-related PTs) were identified.

### 2.4 Statistical analysis

To detect drug-related AE signals, four disproportionality analysis methods were employed: two non-Bayesian (reporting odds ratio [ROR] and proportional reporting ratio [PRR]) and two Bayesian (Bayesian Confidence Propagation Neural Network [BCPNN] and multi-item gamma Poisson shrinker [MGPS]) [26,27]. PRR is a technique for evaluating relative risk, known for its high sensitivity but is prone to false-positive signals, particularly when the number of cases is small [28]. ROR, a more reliable estimator of rate or hazard ratio, offers less bias compared to other metrics [29]. Compared to PRR and ROR, BCPNN has the advantage of delivering relatively stable results, even with a limited number of reports [30]. MGPS is especially effective at identifying signals related to rare or unexpected events [31]. To ensure conservative and reliable results, only those AE signals with at least 3 records that were identified as significant by all four disproportionality analysis methods (ROR, PRR, BCPNN, and MGPS) were considered positive and included in the final analysis [30,32–34]. By combining ROR, PRR, BCPNN, and MGPS, this study leverages the strengths of each method. This integrated approach allows for more efficient identification of ADE signals, minimizes the occurrence of false positives through cross-validation, and enhances the identification of rare adverse reactions by regulating variances and thresholds [30,32,33]. Calculations for all four methods are based on contingency table data (see Table 1), with Table 2 detailing the positive signal thresholds for each method.

**Table 1:** The two-by-two contingency table for disproportionality analysis.

	Target ADEs	Non-target ADEs	Total
**Definity**	a	b	a + b
**Non-Definity**	c	d	c + d
**Total**	a + c	b + d	N = a + b + c + d

Note: a: The number of reports involving the target drug and its associated target adverse drug event. b: The number of reports involving the target drug but associated with other adverse drug events. c: The number of reports involving other drugs but associated with the target adverse drug event. d: The number of reports involving other drugs and their associated other adverse drug events.

**Table 2:** The detailed formulas and thresholds for ROR, PRR, BCPNN, and MGPS methods.

Methods	Formula	Threshold
ROR	ROR = $\frac{\frac{a}{c}}{\frac{b}{d}}$ = $\frac{ad}{bc}$	a ≥ 3 and 95% Cl (lower limit) > 1
	SE(lnROR) =$\;\sqrt{\frac{1}{a}+\frac{1}{b}+\frac{1}{c}+\frac{1}{d}}$	
	95% CI = $e^{\text{ln}(ROR)\pm \text{1}.\text{96}\sqrt{\frac{1}{a}+\frac{1}{b}+\frac{1}{c}+\frac{1}{d}}}$	
PRR	PRR = $\frac{\frac{a}{\left(a+b\right)}}{\frac{c}{\left(c+d\right)}}$ = $\frac{a(c+d)}{c(a+b)}$	a ≥ 3 and 95% Cl (lower limit) > 1
	SE(lnPRR) = $\sqrt{\frac{1}{a}-\frac{1}{\left(a+b\right)}+\frac{1}{c}-\frac{1}{\left(c+d\right)}}$	
	95% CI = $e^{\text{ln}(PRR)\pm \text{1}.\text{96}\sqrt{\frac{1}{a}-\frac{1}{\left(a+b\right)}+\frac{1}{c}-\frac{1}{\left(c+d\right)}}}$	
BCPNN	IC = ${\text{log}}_{2}\frac{p(x,y)}{p(x)p(y)}$ = ${\text{log}}_{2}\frac{a(a+b+c+d)}{\left(a+b)(a+c\right)}$	IC025 > 0
	E(IC) = ${\text{log}}_{2}\frac{\left(a+\gamma 11)(a+b+c+d+\alpha )(a+b+c+d+\beta \right)}{\left(a+b+c+d+\gamma )(a+b+\alpha 1)(a+b+\beta 1\right)}$	
	V(IC) = $\frac{1}{{\left(\text{ln2}\right)}^{2}}\left\{\left[\frac{(a+b+c+d)-a+\gamma -\gamma 11}{\left(a+\gamma 11)(1+a+b+c+d+\gamma \right)}\right]+\left[\frac{(a+b+c+d)-(a+b)+\alpha -\alpha 1}{\left(a+b+\alpha 1)(1+a+b+c+d+\alpha \right)}\right]+\left[\frac{(a+b+c+d)-(a+c)+\beta -\beta 1}{\left(a+c+\beta 1)(1+a+b+c+d+\beta \right)}\right]\right\}$	
	γ = γ11$\frac{\left(a+b+c+d+\alpha )(a+b+c+d+\beta \right)}{\left(a+b+\alpha 1)(a+c+\beta 1\right)}$	
	IC−2SD = E(IC)$-2\sqrt{V(IC)}$	
MGPS	EBGM = $\frac{a(a+b+c+d)}{\left(a+b)(a+c\right)}$	EBGM05 > 2
	95%CI=$e^{\text{ln}(\text{EBGM})\pm \text{1}.\text{96}\sqrt{\frac{1}{a}+\frac{1}{b}+\frac{1}{c}+\frac{1}{d}}}$	

Note: CI: confidence interval; χ2, chi-squared; ROR, reporting odds ratio; PRR, proportional reporting ratio; BCPNN, Bayesian confidence propagation neutral network; IC, information component; IC025, the lower limit of the 95% CI of the IC; E (IC), the IC expectations; V (IC), the variance of IC; EBGM, empirical Bayesian geometric mean; EBGM05, the lower limit of the 95% CI of EBGM; MGPS: multi-item gamma Poisson shrinker.

### 2.5 Sensitivity analysis

To reduce potential bias and enhance the robustness of the results, this study employed both univariate and multivariate logistic regression models to assess the associations of age, body weight, and gender with the risk of Definity-related AEs [35,36]. All three covariates were included in the multivariate model to estimate adjusted odds ratios and 95% confidence intervals. Reports with missing data on any of the covariates were excluded to ensure the validity of the multivariable logistic regression analysis. Given the large number of PTs, we conducted the Bonferroni correction to address the issue of multiple testing and reduce the likelihood of false-positive signals. Results with a P value below 4.07 × 10^−6^ (P < 0.05/12281) were regarded as strong evidence, indicating robust statistical significance. Signals with nominal significance (P < 0.05) that did not maintain significance after Bonferroni correction (P > 4.07 × 10^−6^) were classified as potential signals.

## 3. Results

### 3.1 Descriptive characteristics

In this study, 17559535 reported cases were obtained from the FAERS database during the selected period (from Q1 2004 to Q1 2024), and 4460 ADEs related to Definity and 12281 PTs related to Definity were finally obtained with the removal of the duplicates. The demographic characteristics of ADEs associated with Definity are described in Table 3. The number of reports identifying the gender of the submitters was 4193, with 263 reports (5.9%) missed, 2037 reports submitted by male (45.7%) and 2156 by female (48.4%). 3540 reports possess age-specific information, with 11 (0.2%) reports below 18 years of age, 125 (2.8%) exceeding 85 years of age, 2083 (46.7%) between 18 and 64.9, and 1321 (29.6%) between 65 and 85, respectively. Weight data were available for 2410 patients, with the group between 50–100 kg accounting for the largest proportion (1352, 20.20%). The main reporters are consumers (3022, 67.8%), followed by other healthcare professionals (670, 15.0%), healthcare professionals (454, 10.2%), pharmacist (130, 2.9%) and medical doctor (118, 2.6%). In addition, of all the countries, the United States has reported the highest number of cases, accounting for more than 95%. In terms of outcomes of recorded AEs, the most common serious outcome was hospitalization (212, 4.8%), followed by life-threatening (150, 3.4%), death (75, 1.7%) and disability (10, 0.2%). In addition, 70.02% of AEs occurred within 0–30 days following Definity administration.

**Table 3:** The demographic characteristics of ADEs reported in the FAERS database (January 2004-March 2024) with Definity as the primary suspect drug.

Characteristics	Categorization	The number of Cases	The proportion of Cases
Gender	Female	2156	48.40%
	Male	2037	45.70%
	Unknown	263	5.90%
AGE	<18 years	11	0.20%
	18-64.9 years	2083	46.70%
	65-85 years	1321	29.60%
	>85 years	125	2.80%
	Unknown	916	20.60%
Weight	<50 kg	46	1.00%
	50 ~ 100 kg	1352	30.30%
	>100 kg	1012	22.70%
	Unknown	2046	45.90%
Reported person	Consumers	3022	67.80%
	Healthcare professionals	454	10.20%
	Medical doctor	118	2.60%
	Other healthcare professionals	670	15.00%
	Pharmacist	130	2.90%
	Registered nurse	1	0.00%
	Unknown	61	1.40%
Reported country	Australia	3	0.07%
	Brazil	1	0.02%
	Canada	7	0.16%
	Country Not Specified	8	0.18%
	France	3	0.07%
	Germany	1	0.02%
	Puerto Rico	3	0.07%
	United States of America	4286	96.18%
	Unknown	144	3.23%
Outcome	Death	75	1.70%
	Disability	10	0.20%
	Hospitalization	212	4.80%
	life-threatening	150	3.40%
	Other serious outcomes	800	18.00%
	Required intervention	7	0.20%
	Unknown	3202	71.90%
Time	0-30 days	3120	70.02%
	31-60 days	2	0.04%
	61-90 days	1	0.02%
	91-120 days	1	0.02%
	121-180days	1	0.02%
	181-360days	2	0.04%
	>360 days	6	0.13%
	Unknown	1323	29.69%

Note: FAERS: Food and Drug Administration (FDA) Adverse Event Reporting System; ADE, adverse drug event.

**Fig 2:**
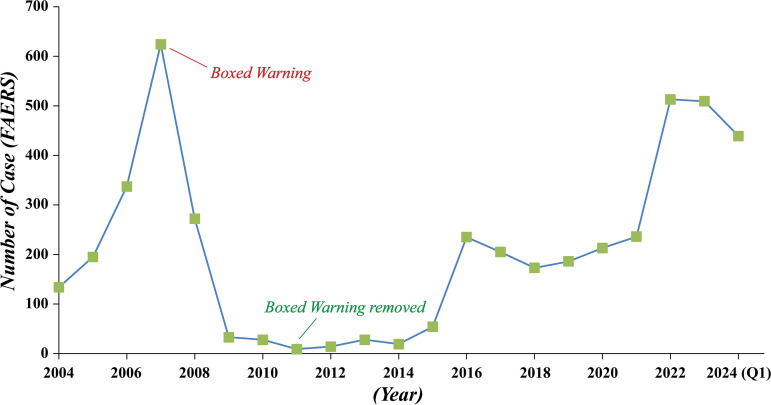
The Number of ADE reports submitted on an annual basis in relation to Definity. FAERS, Food and Drug Administration (FDA) Adverse Event Reporting System (FAERS).

The annual number of ADE reports associated with Definity is presented in Fig 2. The number of reports increased steadily from 2004 (134 reports), reaching a peak in 2007 with 624 reports. This surge may be attributed to both the drug’s expanding clinical use and increased regulatory attention during that period. A subsequent sharp decline was observed, with reports dropping to just 9 in 2011. This may be due to the 2007 FDA label change that introduced a boxed warning, which likely led to reduced use and increased caution among clinicians. Following the removal of the boxed warning in 2011, reporting began to rise again, reaching 235 cases in 2016. From 2016 to 2021, annual reporting remained relatively stable, ranging between 173 and 236 cases. Notably, a renewed increase occurred from 2022 to 2024, with 513, 509, and 439 reports, respectively. This resurgence may reflect increased clinical use, enhanced pharmacovigilance practices, or greater awareness and reporting of AEs. Overall, these fluctuations are likely due to a combination of evolving regulatory guidelines, changes in prescribing patterns, clinical demand, and improved surveillance mechanisms.

### 3.2 Signal detects at the SOC level

**Fig 3:**
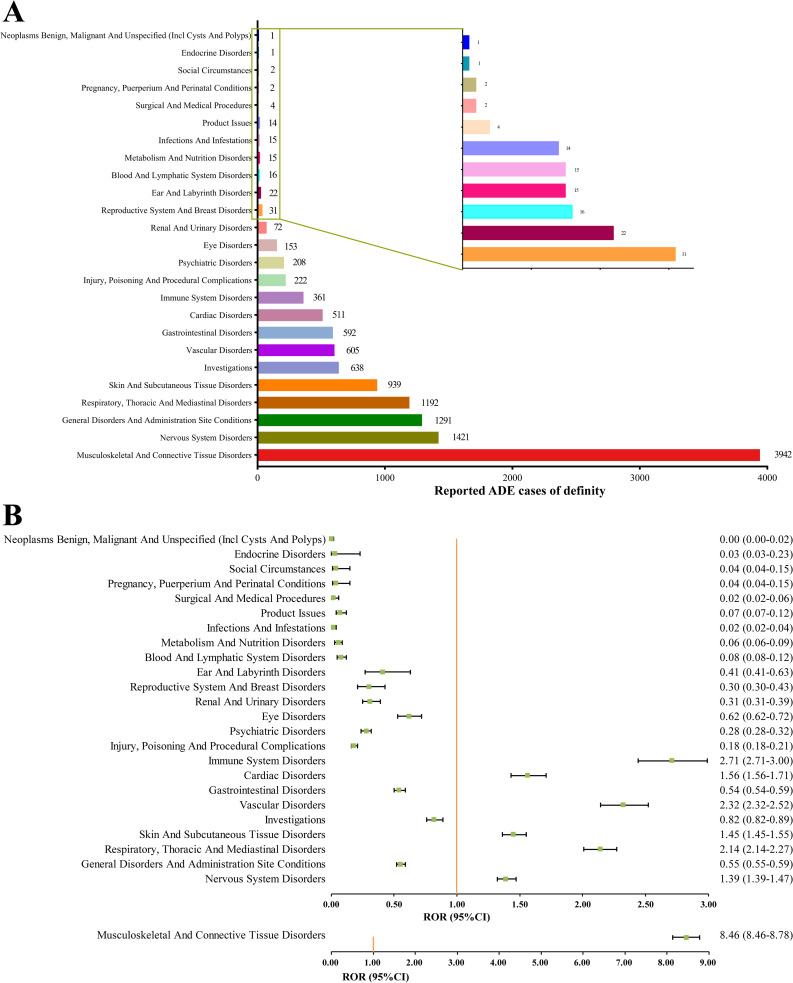
The signals detection at the SOC level. A: The bar chart displaying the number of reported cases of ADEs at each SOC level. B: The ROR values and 95% CI of signals detection at the SOC level. ADE, adverse drug event; SOC, System Organ Class; ROR, reporting odds ratio; CI: confidence interval.

The AE reports and signal strengths of Definity at the SOC level are accessible in Table 4. The ADEs correlated to Definity involved 25 SOCs, with the count of case reports listed in Fig 3A. The top five SOCs in terms of number of reports were musculoskeletal and connective tissue disorders (SOC, N = 3942, 32.10%), nervous system disorders (SOC, N = 1421, 11.57%), general disorders and administration site conditions (SOC, N = 1291, 10.51%), respiratory thoracic and mediastinal disorders (SOC, N = 1192, 9.71%), skin and subcutaneous tissue disorders (SOC, N = 939, 7.65%). The significant SOCs that meet the criteria for at least one of the four disproportionality analysis methods are musculoskeletal and connective tissue disorders (SOC, N = 3942), nervous system disorders (SOC, N = 1421), respiratory, thoracic and mediastinal disorders (SOC, N = 1192), skin and subcutaneous tissue disorders (SOC, N = 939), vascular disorders (SOC, N = 605), cardiac disorders (SOC, N = 511), and immune system disorders (SOC, N = 361). It was worth noting that only one SOC, musculoskeletal and connective tissue disorders met all four disproportionality analysis methods. The RORs for SOC signal intensities linked to Definity are shown in Fig 3B.

**Table 4:** Signal strengths and reports of Definity at the SOC level.

SOC name	Frequency	Reported Case (n)	ROR (95%CI)	PRR (95%CI)	χ²	IC (IC025)	EBGM (EBGM05)
Musculoskeletal And Connective Tissue Disorders	32.13%	3942	8.46 (8.14-8.78)	6.06 (6.04-6.09)	17578.46	2.60 (0.93)	6.06 (5.87)
General Disorders and Administration Site Conditions	10.52%	1291	0.55 (0.52-0.59)	0.60 (0.55-0.65)	414.34	−0.73 (−2.4)	0.60 (0.57)
Nervous System Disorders	11.58%	1421	1.39 (1.32-1.47)	1.35 (1.30-1.40)	140.26	0.43 (−1.23)	1.35 (1.29)
Investigations	5.20%	638	0.82 (0.76-0.89)	0.83 (0.76-0.91)	22.78	−0.26 (−1.93)	0.83 (0.78)
Cardiac Disorders	4.16%	511	1.56 (1.43-1.71)	1.54 (1.45-1.62)	98.95	0.62 (−1.04)	1.54 (1.43)
Immune System Disorders	2.94%	361	2.71 (2.44-3.00)	2.66 (2.55-2.76)	376.47	1.41 (−0.26)	2.65 (2.43)
Vascular Disorders	4.93%	605	2.32 (2.14-2.52)	2.26 (2.18-2.33)	431.92	1.17 (−0.49)	2.25 (2.11)
Respiratory, Thoracic and Mediastinal Disorders	9.71%	1192	2.14 (2.01-2.27)	2.03 (1.97-2.08)	650.21	1.02 (−0.65)	2.03 (1.93)
Skin And Subcutaneous Tissue Disorders	7.65%	939	1.45 (1.36-1.55)	1.42 (1.36-1.48)	123.28	0.51 (−1.16)	1.42 (1.34)
Gastrointestinal Disorders	4.82%	592	0.54 (0.50-0.59)	0.56 (0.48-0.64)	221.53	−0.83 (−2.5)	0.56 (0.52)
Product Issues	0.11%	14	0.07 (0.04-0.12)	0.07 (0.45-0.6)	164.99	−3.76 (−5.42)	0.07 (0.05)
Injury, Poisoning and Procedural Complications	1.81%	222	0.18 (0.16-0.21)	0.20 (−0.07-0.33)	794.17	−2.34 (−4.00)	0.2 (0.18)
Renal And Urinary Disorders	0.59%	72	0.31 (0.25-0.39)	0.31 (0.08-0.54)	110.1	−1.67 (−3.34)	0.31 (0.26)
Psychiatric Disorders	1.70%	208	0.28 (0.24-0.32)	0.29 (0.16-0.43)	379.26	−1.78 (−3.44)	0.29 (0.26)
Eye Disorders	1.25%	153	0.62 (0.53-0.72)	0.62 (0.46-0.78)	35.93	−0.69 (−2.35)	0.62 (0.54)
Ear And Labyrinth Disorders	0.18%	22	0.41 (0.27-0.63)	0.41 (0.00-0.83)	18.43	−1.28 (−2.94)	0.41 (0.29)
Infections And Infestations	0.12%	15	0.02 (0.01-0.04)	0.02 (−0.48-0.53)	648.24	−5.42 (−7.09)	0.02 (0.02)
Metabolism And Nutrition Disorders	0.12%	15	0.06 (0.03-0.09)	0.06 (−0.45-0.56)	241.82	−4.15 (−5.81)	0.06 (0.04)
Reproductive System and Breast Disorders	0.25%	31	0.30 (0.21-0.43)	0.30 (−0.05-0.65)	50.7	−1.73 (−3.4)	0.30 (0.22)
Neoplasms Benign, Malignant and Unspecified (Incl Cysts and Polyps)	0.01%	1	0.00 (0.00-0.02)	0.00 (−1.96-1.96)	340.64	−8.38 (−10.05)	0.00 (0.00)
Blood And Lymphatic System Disorders	0.13%	16	0.08 (0.05-0.12)	0.08 (−0.41-0.57)	181.88	−3.71 (−5.38)	0.08 (0.05)
Social Circumstances	0.02%	2	0.04 (0.01-0.15)	0.04 (−1.35-1.42)	49.25	−4.73 (−6.39)	0.04 (0.01)
Surgical And Medical Procedures	0.03%	4	0.02 (0.01-0.06)	0.02 (−0.96-1.00)	158.77	−5.36 (−7.03)	0.02 (0.01)
Pregnancy, Puerperium and Perinatal Conditions	0.02%	2	0.04 (0.01-0.15)	0.04 (−1.35-1.42)	50.12	−4.75 (−6.42)	0.04 (0.01)
Endocrine Disorders	0.01%	1	0.03 (0.00-0.23)	0.03 (−1.93-1.99)	29.16	−4.96 (−6.62)	0.03 (0.01)

Note: SOC, system organ class; n, the number of reports; ROR, reporting odds ratio; CI: confidence interval; PRR, proportional reporting ratio; χ2, chi-squared; IC, information component; IC025, the lower limit of the 95% CI of the IC; EBGM, empirical Bayesian geometric mean; EBGM05, the lower limit of the 95% CI of EBGM.

### 3.3 Disproportionality analysis for ADEs associated with Definity use

After excluding possible indications for Definity use, a total of 104 significant PTs were found that satisfied all four Disproportionality analysis, detailed in S2 Table. We identified that PT entries with more than 100 cases included back pain (PT, n = 1979), muscle spasms (PT, n = 570), dyspnoea (PT, n = 562), pain in extremity (PT, n = 418), flushing (PT, n = 327), chest pain (PT, n = 308), urticaria (PT, n = 272), neck pain (PT, n = 230), blood pressure increased (PT, n = 160), erythema (PT, n = 156), anaphylactic reaction (PT, n = 146), chest discomfort (PT, n = 129), heart rate increased (PT, n = 117), musculoskeletal pain (PT, n = 116), feeling hot (PT, n = 115), referring to Table 5. Interestingly, unexpected significant ADEs were identified, and PTs with more than 20 reports included eye movement disorder (PT), renal pain (PT). Notably, the vast majority of these signals remained statistically significant after Bonferroni correction (P < 4.07 × 10^−6^). Only 21 signals did not maintain significance after Bonferroni correction: cold sweat (PT), sinus tachycardia (PT), dizziness postural (PT), screaming (PT), acute pulmonary oedema (PT), blood pressure systolic decreased (PT), electrocardiogram ST segment elevation (PT), extravasation (PT), testicular pain (PT), anaphylactoid reaction (PT), choking sensation (PT), throat clearing (PT), oedema mouth (PT), staring (PT), pulmonary pain (PT), blood pressure immeasurable (PT), foaming at mouth (PT), pharyngeal paraesthesia (PT), electric shock sensation (PT), immediate post-injection reaction (PT) and moaning (PT).

**Table 5:** The top 15 PTs satisfying all four disproportionality analysis.

SOC name	PT name	Case number (n)	ROR (95%Cl)	P_-ROR_	PRR (95%CI)	P_-PRR_	P_-Bon_	χ2	EBGM (EBGM05)	IC (IC025)
Musculoskeletal And Connective Tissue Disorders	Back Pain	1979	49.27 (46.94-51.71)	1.00E-300	41.49 (41.45-41.53)	1.00E-300	4.81E-04	77728	41.09 (39.46)	5.36 (3.69)
Musculoskeletal And Connective Tissue Disorders	Muscle Spasms	570	15.71 (14.44-17.09)	1.00E-300	15.02 (14.94-15.10)	1.00E-300	4.81E-04	7458	14.97 (13.95)	3.9 (2.24)
Respiratory, Thoracic and Mediastinal Disorders	Dyspnoea	562	5.03 (4.62-5.48)	1.00E-300	4.85 (4.77-4.93)	1.00E-300	4.81E-04	1731	4.84 (4.51)	2.28 (0.61)
Musculoskeletal And Connective Tissue Disorders	Pain In Extremity	418	6.92 (6.28-7.63)	1.00E-300	6.72 (6.63-6.81)	1.00E-300	4.81E-04	2042	6.71 (6.18)	2.75 (1.08)
Vascular Disorders	Flushing	327	15.44 (13.83-17.24)	1.00E-300	15.06 (14.95-15.17)	1.00E-300	4.81E-04	4284	15.01 (13.69)	3.91 (2.24)
General Disorders and Administration Site Conditions	Chest Pain	308	8.09 (7.22-9.06)	1.00E-300	7.91 (7.80-8.02)	1.00E-300	4.81E-04	1861	7.90 (7.18)	2.98 (1.31)
Skin And Subcutaneous Tissue Disorders	Urticaria	272	8.47 (7.51-9.56)	1.00E-300	8.31 (8.19-8.43)	1.00E-300	4.81E-04	1750	8.29 (7.50)	3.05 (1.39)
Musculoskeletal And Connective Tissue Disorders	Neck Pain	230	20.55 (18.03-23.42)	1.00E-300	20.19 (20.06-20.31)	1.00E-300	4.81E-04	4178	20.09 (18.01)	4.33 (2.66)
Investigations	Blood Pressure Increased	160	5.14 (4.4-6.01)	1.00E-300	5.09 (4.93-5.24)	1.00E-300	4.81E-04	525.8	5.08 (4.46)	2.34 (0.68)
Skin And Subcutaneous Tissue Disorders	Erythema	156	3.74 (3.19-4.38)	1.00E-300	3.70 (3.55-3.86)	1.00E-300	4.81E-04	308.6	3.70 (3.24)	1.89 (0.22)
Immune System Disorders	Anaphylactic Reaction	146	13.99 (11.88-16.47)	1.00E-300	13.83 (13.67-13.99)	1.00E-300	4.81E-04	1734	13.79 (12.03)	3.79 (2.12)
General Disorders and Administration Site Conditions	Chest Discomfort	129	6.37 (5.35-7.57)	1.00E-300	6.31 (6.14-6.48)	1.00E-300	4.81E-04	576.5	6.30 (5.45)	2.66 (0.99)
Investigations	Heart Rate Increased	117	5.84 (4.87-7.01)	1.00E-300	5.79 (5.61-5.97)	1.00E-300	4.81E-04	464.1	5.79 (4.97)	2.53 (0.87)
Musculoskeletal And Connective Tissue Disorders	Musculoskeletal Pain	116	10.06 (8.38-12.08)	1.00E-300	9.98 (9.80-10.16)	1.00E-300	4.81E-04	935.6	9.96 (8.54)	3.32 (1.65)
General Disorders and Administration Site Conditions	Feeling Hot	115	9.09 (7.56-10.92)	1.00E-300	9.01 (8.83-9.20)	1.00E-300	4.81E-04	818.5	9.00 (7.71)	3.17 (1.50)

Note: PT, preferred term; SOC, system organ class; n, the number of reports; ROR, reporting odds ratio; CI: confidence interval; PRR, proportional reporting ratio; χ2, chi-squared; EBGM, empirical Bayesian geometric mean; EBGM05, the lower limit of the 95% CI of EBGM. IC, information component; IC025: the lower limit of the 95% CI of the IC; P_-ROR_: The P value of ROR method; P_-PRR_: The P value of PRR method; P_-Bon_: The P value after Bonferroni correction.

As the most rigorous algorithm, we next sorted these significant PTs using the results of EBGM and listed the top 30 in Table 6 and S3 Table. In this study, agonal respiration (PT, N = 6, ROR: 213.06, PRR: 212.95, IC: 7.66, EBGM: 202.61), seizure like phenomena (PT, N = 19, ROR: 61.26, PRR: 61.16, IC: 5.91, EBGM: 60.29), coccydynia (PT, N = 16, ROR: 56.00, PRR: 55.93, IC: 5.79, EBGM: 55.20), back pain (PT, N = 1979, ROR: 49.27, PRR: 41.49, IC: 5.36, EBGM: 41.09), flank pain (PT, N = 79, ROR: 41.64, PRR: 41.37, IC: 5.36, EBGM: 40.98), etc., were consistent with the ADE in the drug’s instructions. In addition, breath holding (PT, N = 4, ROR: 89.33, PRR: 89.30, IC: 6.45, EBGM: 87.44), vasospasm (PT, N = 6, ROR: 32.42, PRR: 32.40, IC: 5.01, EBGM: 32.16), retrograde amnesia (PT, N = 3, ROR: 29.11, PRR: 29.11, IC: 4.85, EBGM: 28.91), scrotal pain (PT, N = 3, ROR: 25.91, PRR: 25.90, IC: 4.69, EBGM: 25.75), were not mentioned in the drug’s leaflet, and warrants further investigation.

**Table 6:** The PTs with EBGM>25.00.

SOC name	PT name	Case number (n)	ROR (95%Cl)	P_-ROR_	PRR (95%CI)	P_-PRR_	P_-Bon_	χ2	EBGM (EBGM05)	IC (IC025)
Respiratory, Thoracic and Mediastinal Disorders	Agonal Respiration	6	213.06 (93.78-484.04)	1.00E-300	5.04 (4.24-5.84)	1.00E-300	4.81E-04	1204	202.61 (101.97)	7.66 (5.98)
Psychiatric Disorders	Breath Holding	4	89.33 (33.17-240.57)	1.00E-300	5.28 (4.30-6.26)	1.00E-300	4.81E-04	341.9	87.44 (38.17)	6.45 (4.77)
Immune System Disorders	Contrast Media Reaction	33	69.25 (49.07-97.73)	1.00E-300	69.07 (68.73-69.41)	1.00E-300	4.81E-04	2177.65	67.96 (50.94)	6.09 (4.42)
Nervous System Disorders	Seizure Like Phenomena	19	61.26 (38.93-96.38)	1.00E-300	61.16 (60.71-61.61)	1.00E-300	4.81E-04	1108.09	60.29 (41.26)	5.91 (4.25)
Musculoskeletal And Connective Tissue Disorders	Coccydynia	16	56.00 (34.18-91.74)	1.00E-300	4.40 (3.91-4.89)	1.00E-300	4.81E-04	851.66	55.2 (36.52)	5.79 (4.12)
Musculoskeletal And Connective Tissue Disorders	Back Pain	1979	49.27 (46.94-51.71)	1.00E-300	41.49 (41.45-41.53)	1.00E-300	4.81E-04	77727.64	41.09 (39.46)	5.36 (3.69)
Musculoskeletal And Connective Tissue Disorders	Flank Pain	79	41.64 (33.34-52)	1.00E-300	41.37 (41.15-41.59)	1.00E-300	4.81E-04	3082.22	40.98 (34.02)	5.36 (3.69)
Vascular Disorders	Vasospasm	6	32.42 (14.51-72.39)	1.00E-300	32.40 (31.60-33.20)	1.00E-300	4.81E-04	181.17	32.16 (16.42)	5.01 (3.34)
Nervous System Disorders	Retrograde Amnesia	3	29.11 (9.35-90.64)	5.97E-09	11.15 (10.02-12.29)	1.00E-300	4.81E-04	80.86	28.91 (11.18)	4.85 (3.18)
Cardiac Disorders	Pulseless Electrical Activity	23	28.27 (18.76-42.62)	1.00E-300	28.22 (27.81-28.63)	1.00E-300	4.81E-04	599.92	28.04 (19.89)	4.81 (3.14)
Musculoskeletal And Connective Tissue Disorders	Groin Pain	48	26.22 (19.73-34.84)	1.00E-300	26.12 (25.84-26.40)	1.00E-300	4.81E-04	1152.53	25.96 (20.47)	4.7 (3.03)
Reproductive System and Breast Disorders	Scrotal Pain	3	25.91 (8.32-80.62)	1.94E-08	9.23 (8.10-10.36)	1.00E-300	4.81E-04	71.37	25.75 (9.96)	4.69 (3.02)

Note: PT, preferred term; EBGM, empirical Bayesian geometric mean; SOC, system organ class; n, the number of reports; ROR, reporting odds ratio; CI: confidence interval; PRR, proportional reporting ratio; χ2, chi-squared; EBGM05, the lower limit of the 95% CI of EBGM. IC, information component; IC025: the lower limit of the 95% CI of the IC; P-ROR: The P value of ROR method; P-PRR: The P value of PRR method; P-Bon: The P value after Bonferroni correction.

### 3.3 Logistic regression analysis

The associations between patient gender, age, and body weight and the top 15 AEs related to Definity are presented in S4 Table. Our analysis indicated that gender and body weight were important factors influencing multiple AEs. Multivariate logistic regression revealed that male patients had a significantly lower risk of experiencing dyspnea (PT), pain in extremities (PT), flushing (PT), chest pain (PT), neck pain (PT), increased heart rate (PT), and feeling hot (PT) compared to female patients (all P < 0.05). In contrast, the risks of urticaria (PT) and anaphylactic reactions (PT) were significantly higher in males. In addition, individuals weighing ≥50 kg had a significantly increased risk of back pain (PT, OR: 2.10, 95% CI: 1.09–4.31, P = 0.032), and a reduced risk of increased heart rate (PT, OR: 0.31, 95% CI: 0.12–0.99, P = 0.036). In contrast, age was not significantly associated with the majority of AEs.

## 4. Discussion

This study aimed to evaluate ADEs associated with Definity, an ultrasound contrast agent, using real-world data from the FAERS database over the period from 2004 to the first quart of 2024. A total of 104 statistically significant signals were identified at the PT level, and the vast majority remained significant following Bonferroni correction. These signals included both established adverse reactions and new safety concerns about Definity that warrant further attention. Notably, we observed previously under-recognized signals related to pain and eye disorders. These findings underscore the importance of continued pharmacovigilance to monitor and address potential risks associated with Definity, and provide a foundation for enhancing risk communication, informing regulatory decision-making, and updating clinical practice guidelines.

Our findings extend the early pharmacovigilance insights reported by Main et al., who examined post-marketing safety data for Definity and highlighted back pain and flank pain as key AEs [37]. Our analysis builds upon this foundational work by utilizing a more comprehensive and updated dataset, along with multiple signal detection algorithms. In doing so, we identified additional potential safety signals—such as eye disorders—that were not emphasized in the 2015 study. These differences may reflect improvements in ADE recognition, evolving clinical usage patterns, or changes in post-marketing reporting behavior over time.

Our analysis revealed that musculoskeletal and connective tissue disorders were the most frequently reported SOC associated with Definity use. At the PT level, back pain (PT), muscle spasms (PT), and pain in extremity (PT) were the most commonly observed ADEs. The incidence of these disorders was significantly higher than that of other SOCs, reinforcing previous findings in the literature and aligning with the prescribing information for Definity. This may be explained by the pressure exerted by Definity injections on blood vessels and surrounding tissues, which can induce localized inflammatory responses leading to muscle pain and discomfort [38]. The potential underlying mechanism involves the interaction of microbubbles with the vascular endothelium [39]. Upon injection, the microbubbles may cause mechanical stress on the vessel walls, triggering inflammatory cascades that result in tissue pain. These responses may include the release of cytokines and other mediators that contribute to muscle soreness following administration [40]. Recognizing these associations is crucial, as it underscores the importance of informing patients about possible musculoskeletal reactions and closely monitoring them post-injection. In addition to musculoskeletal events, we identified significant signals involving the nervous, cardiovascular, and respiratory systems, including respiratory arrest (PT), vasospasm (PT), and seizure-like phenomena (PT). These serious ADEs highlight the need for increased clinical vigilance, particularly in patients with pre-existing cardiac or pulmonary conditions, who may be at greater risk. Clinicians should be prepared to intervene promptly in response to any acute reactions following Definity administration. Our findings are generally consistent with FDA safety communications, including the 2007 warning on cardiopulmonary risks and the 2021 advisory concerning allergic reactions in patients sensitive to polyethylene glycol. Signals such as dyspnea, chest discomfort, and allergic reactions were prominent in our analysis, supporting current recommendations to screen high-risk patients prior to use. In addition, we detected several previously unexpected ADEs reported more than 20 times, such as eye movement disorder (PT) and renal pain (PT). Eye movement disorders could potentially be associated with central nervous system microvascular effects, which have been occasionally observed with intravenous microbubble contrast agents [41]. Although not extensively studied in humans, preclinical evidence suggests that complement activation induced by microbubbles might transiently alter vascular permeability or neural signaling in sensitive regions, including the brainstem or cranial nerves [12]. As for renal pain (PT), prior studies have indicated that lipid-shelled microbubbles like Definity may be retained in the renal microcirculation—particularly in the cortical region—where they could generate complement-related intermediates known to mediate nociceptive responses [42]. While these proposed mechanisms remain speculative, they suggest possible biologic plausibility and highlight the need for further mechanistic and clinical research to better understand these associations.

A noteworthy finding in our study is that the majority of AEs occurred within the first 30 days following Definity administration, suggesting that most reactions tend to arise shortly after exposure. This time pattern highlights the importance of close observation during the early post-administration period, particularly for patients with known risk factors. Although nearly 30% of reports lacked valid onset information due to limitations in data structure, the clear clustering of AEs within the initial month provides actionable insight for clinical monitoring strategies.

Our study found that male patients had a significantly lower risk of experiencing dyspnea (PT), pain in extremities (PT), flushing (PT), chest pain (PT), neck pain (PT), increased heart rate (PT), and feeling hot (PT) compared to female patients (all P < 0.05). In contrast, the risks of urticaria (PT) and anaphylactic reactions (PT) were significantly higher in males. These observations may be attributed to several factors, including differences in body composition, hormonal regulation, and genetic predisposition [43–46]. For example, males and females may metabolize drugs at different rates due to sex-based differences in hepatic enzyme activity, which can influence systemic drug concentrations and pharmacodynamic responses [47,48]. Additionally, hormonal fluctuations in females—particularly related to estrogen and progesterone—may modulate immune responses and alter susceptibility to certain ADEs [46]. Moreover, the observed increase in back pain (PT) risk and the reduction in heart rate elevation (PT) among individuals weighing ≥50 kg may be explained by differences in drug pharmacokinetics, increased mechanical loading on the musculoskeletal system, and altered autonomic responses in heavier individuals [49,50]. However, these findings should be interpreted with caution due to potential sample size limitations and residual confounding. Overall, the identification of sex- and weight-related differences in ADE occurrence underscores the importance of individualized approaches in clinical decision-making. Tailoring treatment protocols based on patient sex and body weight may enhance both safety and therapeutic efficacy. Future clinical studies are encouraged to stratify analyses by sex to better understand these differences and their implications for precision medicine.

Moreover, in clinical, it is important to contextualize the identified AE signals when considering the risk-benefit profile of Definity. Several detected signals, such as transient back pain (PT), renal pain (PT) and muscle spasms (PT), appear to be non-life-threatening and self-resolving. These reactions should be communicated transparently before Definity injection to ensure informed consent and reduce anxiety. At the same time, the diagnostic enhancement offered by Definity, especially in patients with suboptimal echocardiographic windows, can be critical in guiding appropriate treatment. Thus, while vigilance and appropriate monitoring are warranted, particularly for patients with a history of hypersensitivity reactions, the occurrence of mild and transient symptoms should be carefully weighed against the agent’s significant diagnostic utility.

In light of these findings, it is also crucial to acknowledge the limitations of our current study. Although the FAERS database serves as a valuable tool for pharmacovigilance, its dependence on a voluntary reporting system introduces inherent biases. Issues such as underreporting and selective reporting may result in AEs not being consistently or comprehensively captured, particularly when they are perceived as less severe or unlikely to be drug-related. These limitations can compromise the completeness and accuracy of the data. Furthermore, several well-recognized reporting biases intrinsic to spontaneous reporting systems may have influenced our results. For example, the Weber effect—a phenomenon in which AE reports surge shortly after a drug’s market introduction and then decline—may have contributed to the observed early peak in reports [51]. The notoriety bias, where media coverage or regulatory warnings increase the likelihood of specific AE reporting, may also explain temporal spikes for certain events, such as cardiopulmonary symptoms following the 2007 FDA boxed warning on Definity [52]. Additionally, masking bias could have obscured weaker signals due to the dominance of more frequently reported AEs, thereby influencing the sensitivity of disproportionality analysis [53]. Moreover, the lack of standardized and complete data on indications means that reports involving off-label uses could not be reliably excluded, potentially introducing additional confounding factors. FAERS also lacks detailed clinical information, including drug dosage, administration route, timing, and exposure duration, which severely limits our ability to assess causality or explore dose-response relationships. Moreover, the FAERS database does not contain information on the number of patients exposed to Definity or its usage volume over time, which limits the ability to interpret trends in AE reporting as true changes in risk. Observed fluctuations may instead reflect variations in reporting behavior, regulatory actions, or clinical practice patterns, rather than changes in the actual incidence of AEs. Furthermore, echocardiographic contrast agents like Definity are commonly administered to patients with acute illness or complex comorbidities, especially those with underlying cardiovascular conditions. These clinical contexts inherently elevate the background risk for adverse outcomes, making it difficult to distinguish between events caused by the drug itself and those arising from the underlying disease. Therefore, while disproportionality analysis is a valuable tool for signal detection, it is important to emphasize that spontaneous AE reports alone cannot establish causality. These findings should be regarded as exploratory and interpreted with caution, given the inherent limitations of such data sources in inferring direct causal relationships between drug exposure and AEs. Although several novel AEs, such as eye movement disorders and renal pain, were identified as statistically significant, these signals often relied on a small number of reports and lack supporting clinical or mechanistic evidence. Therefore, these findings should be interpreted with caution and viewed as hypothesis-generating rather than confirmatory. Future studies are needed to validate these signals through independent datasets, assess biological plausibility, and explore causal inference using rigorous pharmacoepidemiologic approaches.

To address these limitations, future research should prioritize prospective cohort studies and randomized controlled trials to validate the current findings and investigate the underlying mechanisms of the observed ADEs. These studies ought to include diverse patient populations to improve generalizability and assess the influence of demographic factors, including age, sex, and comorbidities, on the safety profile of Definity. Longitudinal designs that track patients over time following Definity administration may yield critical insights into its long-term safety and the potential for delayed adverse reactions. Furthermore, exploring the biological pathways associated with the reported ADEs could enhance our understanding of the risks and inform more effective risk management strategies.

## 5. Conclusion

In conclusion, this study offers an exploratory, real-world assessment of AEs associated with Definity using FAERS data. While Definity is generally well tolerated, the observed AEs signals, particularly those involving the musculoskeletal, nervous, and respiratory systems, underscore the need for ongoing pharmacovigilance and clinical vigilance. Notably, several emerging signals, such as eye movement disorder and renal pain, are not currently listed in the drug label and should be interpreted with caution as hypothesis-generating observations. Clinicians should remain aware of the potential for AEs and be prepared to monitor and manage them appropriately when using Definity. Although causality cannot be inferred from spontaneous reports, these findings provide a valuable foundation for further investigation and signal validation. The continued integration of pharmacovigilance data into clinical practice will be essential for optimizing patient safety and informing risk management strategies.

## Supporting information

S1 File **S1 Table.** All searched drug names about Definity from the NCBI MeSH database. **S2 Table.** All the PTs satisfying all four Disproportionality analysis. Note: PT, preferred term; SOC, system organ class; n, the number of reports; ROR, reporting odds ratio; CI: confidence interval; PRR, proportional reporting ratio; χ2, chi-squared; EBGM, empirical Bayesian geometric mean; EBGM05, the lower limit of the 95% CI of EBGM. IC, information component; IC025: the lower limit of the 95% CI of the IC; P-ROR: The P value of ROR method; P-PRR: The P value of PRR method; P-Bon: The P value after Bonferroni correction. **S3 Table.** The top 30 PTs according to EBGM. Note: PT, preferred term; EBGM, empirical Bayesian geometric mean; SOC, system organ class; n, the number of reports; ROR, reporting odds ratio; CI: confidence interval; PRR, proportional reporting ratio; χ2, chi-squared; EBGM05, the lower limit of the 95% CI of EBGM. IC, information component; IC025: the lower limit of the 95% CI of the IC; P-ROR: The P value of ROR method; P-PRR: The P value of PRR method; P-Bon: The P value after Bonferroni correction. **S4 Table.** Univariate and multivariate logistic regression analysis of the odds ratio for the top 15 PT of Definity. Note: PT, preferred term; OR, odds ratio; CI: confidence interval.
(ZIP)
